# A Pilot Study of Urokinase-Type Plasminogen Activator (uPA) Overexpression in the Brush Cytology of Patients with Malignant Pancreatic or Biliary Strictures

**DOI:** 10.1155/2009/805971

**Published:** 2009-11-30

**Authors:** John F. Gibbs, Michael Schlieman, Paramvir Singh, Rakhee Saxena, Maisie Martinick, Alan D. Hutson, James Corasanti

**Affiliations:** Roswell Park Cancer Institute, State University of New York at Buffalo, Buffalo, NY 14263, USA

## Abstract

We have previously demonstrated that uPA is overexpressed in pancreatic tumors. In an attempt to diagnose these tumors earlier, we sought to determine whether uPA could be identified in endoscopic retrograde cholangiopancreatography obtained brushings in patients with malignant pancreatic and biliary strictures. Secondarily, uPA was measured in the serum of this patient population. uPA overexpression was identified in the cytologic tissue in 8 of 11 patients (72.7%). Serum analysis demonstrated a 2-fold higher concentration of uPA in the pancreaticobiliary cancer patients (1.27 versus 0.56 ng/mL; *P* = .0182). Also, uPA overexpression correlated with serum levels (*P* < .0001). This study confirms that uPA can be detected in the ERCP cytologically obtained tissue and is frequently present in a higher concentration in the serum of pancreaticobiliary cancer patients. A larger sample size will be required to address its value as a sensitive marker for the diagnosis of pancreatic or biliary cancers.

## 1. Introduction

Pancreaticobiliary cancer remains a lethal disease where treatment continues to be a major oncologic challenge. Most patients present with advanced disease in which survival is dismal. For patients diagnosed early with small tumors, surgery offers a chance of cure. In an attempt to diagnose these tumors at an early stage, many studies have evaluated the use of tumor markers, but so far, due to low sensitivity and specificity, the results have not been promising [1].

The high metastatic potential of pancreatic cancer has led researchers to investigate its ability to invade the basement membrane and extracellular matrix (ECM) allowing it access to capillaries and lymphatics. One process that has been shown to be involved in invasion of most types of cancer is the urokinase-type plasminogen activator system [2]. uPA is a serine proteinase that is best known for catalyzing the conversion of inactive plasminogen to the active proteinase plasmin. uPA and its receptor uPAR have been demonstrated to be involved in tumor invasion, growth, and metastasis. Independently or through the activation of plasmin, uPA can degrade ECM, activate matrix metalloproteinases, mediate the release of growth factors (including transforming growth factor *β*, fibroblast growth factor, vascular endothelial growth factor, insulin growth factor, tumor necrosis factor *α*, and hepatocyte growth factor), stimulate cellular migration, induce chemotaxis, and promote angiogenesis [3, 4].

Since the 1980s when uPA was postulated to have a role in tumor invasion, there have been multiple studies evaluating it as a prognostic marker of cancer. It was first studied in breast cancer where it has been shown as an independent prognostic marker for predicting survival second only to lymph node status [5]. Following the initial results in breast cancer, uPA overexpression was shown to confer a worse prognosis in many other cancers including colorectal, esophageal, gastric, hepatocellular, prostate, sarcoma, and head and neck squamous cells among others [6–12].

The importance of the uPA activator system has also been demonstrated in pancreatic cancer [13–18]. The first study to show overexpression of uPA in pancreatic cancer was by Takeuchi et al. in 1993. He demonstrated by immunohistochemical staining that 78% of pancreatic cancers overexpressed uPA and this overexpression correlated with decreased survival [13]. Another study by Cantero et al. in 1997 showed that concomitant overexpression of uPA and its receptor uPAR correlated with shorter survival times [14]. We detected uPA overexpression in pancreatic intraepithelial neoplasia (PanIN) and histologically normal ducts/acini that are in the immediate vicinity of the tumor. uPA was also found in the vessels of tumor stroma suggesting that uPA is in circulation [15], and therefore should be detectable by serum analysis.

One method of diagnosing pancreaticobiliary tumors is endoscopic retrograde cholangiopancreatography (ERCP). It allows further evaluation through imaging of the ductal system and allows to sample pancreatic juice and brush biopsy cytology for further analysis [19]. ERCP has also been studied in small numbers to screen high-risk patients with a family history of pancreatic cancer, and it demonstrated that cytologic brushings showing dysplasia correlate with the pathologic specimen after resection [20, 21]. Despite combination of sampling with brush cytology, fine needle aspiration, and biopsy, the sensitivity of all three combined is only 62%. Most practitioners perform only brush cytology which by itself has a sensitivity of only 30% in the diagnosis of pancreaticobiliary malignancies. 

We hypothesized that molecular markers such as uPA may improve our ability to differentiate malignant from benign pancreaticobiliary strictures and assist us in planning therapeutic strategies. We performed a feasibility study of patients undergoing ERCP for confirmed or suspected pancreaticobiliary cancers to determine whether uPA overexpression could be identified from cytologic brushings. Serum levels of uPA were measured in this patient group and compared to healthy subjects.

## 2. Patients and Methods

### 2.1. Patients

IRB approvals at University of Buffalo and Roswell Park Cancer Institute were obtained. Eleven patients with known or suspected pancreaticobiliary malignant strictures secondary to either pancreatic cancer or cholangiocarcinoma as determined clinically by radiologic studies were consented to have cytologic brushings obtained during ERCP for evaluation of expression of uPA. At ERCP, cytologic brushings of the common bile duct or pancreatic duct were performed. Brushings were obtained in duplicate. The samples were fixed in 70% ethanol and stored in Phosphate buffered saline. One slide was routinely stained for H&E to identify the presence of epithelial cells and the other was stained for uPA in a blinded fashion. Presence and type of cancer were determined by biopsy and reviewed by a pathologist. Serum samples were drawn from these patients. Eleven normal healthy subjects consented to have serum samples drawn for measurement of uPA.

### 2.2. Antibody and Reagents

uPA1 monoclonal antibody directed toward the catalytic domain of the uPA *β*-chain, no. 3689, was obtained from American Diagnostica Inc. (Greenwich, CT). The specifics of the antibody have previously been described [10]. uPA1 (no. 3689) is strictly active-site specific. Reagents used in the immunohistochemical procedure including blocking solution, secondary antibody, and Vectastain Elite ABC reagent were purchased as the R.T.U Vectastain Universal Elite ABC kit (Vector Laboratories, Burlingame, CA). Color-development reagents were purchased as part of a DAB substrate kit for peroxidase (Vector Laboratories, Burlingame, CA). The DAB substrate kit contained the reagents required to make a working solution of 3,3′-diaminobenzidine (DAB) for staining tissue sections. Triton X-100 was purchased from Sigma (St. Louis, Mo, USA).

### 2.3. Immunohistochemistry

uPA expression in cytologic brushings obtained during ERCP was evaluated by immunohistochemistry (IHC). Cells from the brushings were fixed onto slides. Details of the IHC procedure have been previously described [15]. The uPA1 primary antibody was used at a concentration of 20 *μ*g/mL. Cells were blocked with normal horse serum (2.5%) for one hour and incubated overnight at 4° Celsius with the primary antibody. After gently rinsing the cells with Triton-TBS, they were treated with the secondary antibody (R.T.U. Biotinylated Universal Antibody and Anti-Rabbit/Anti-Mouse IgG made in horse), gently washed and treated with Vectastain Elite ABC reagent. Color development was performed using a DAB substrate kit for peroxidase. The slides were then counterstained with hematoxylin and mounted with a coverslip. Normal kidney sections were used as positive controls for uPA. A slide of cytologic brushings was also stained by the same procedure, except that an irrelevant isotype IgG was substituted for the primary antibody as a negative control. Staining intensity for uPA was classified as 0–3+ (0 absent; 1+, weak; 2+, moderate; 3+, strong).

### 2.4. ELISA

Serum levels of uPA were analyzed using an enzyme-linked immunosorbent assay (ELISA). ELISA kits are available commercially to perform these assays. uPA ELISA kits were purchased from American Diagnostica, Inc. (Greenwich, CT). The assays and data calculations were performed according to the manufacturer's instructions.

### 2.5. Statistical Analysis

Differences between groups were analyzed by an exact Wilcoxon rank-sum test. Correlation between IHC grade and serum levels of uPA was estimated with the Spearman rank correlation coefficient. The exact permutation *P* value computed for testing the correlation is zero versus not. All tests were two sided and tested at level alpha = 0.05. Descriptive statistics were given as mean and standard deviations (SD). All tests were carried forth using SAS 9.2, SAS Institute Inc., Cary, NC.

Given *n* = 11 subjects per group and assuming exchangeability under the null hypothesis, the Wilcoxon rank-sum test would be able to detect approximate shift alternatives of roughly 0.6 ng/mL, 1.3 ng/mL, and 1.7 ng/mL given 0.80 power and common standard deviations under the null hypothesis of no difference of 0.5 ng/mL, 1.0 ng/mL, and 1.9 ng/mL, respectively.

## 3. Results

**Figure 1:**
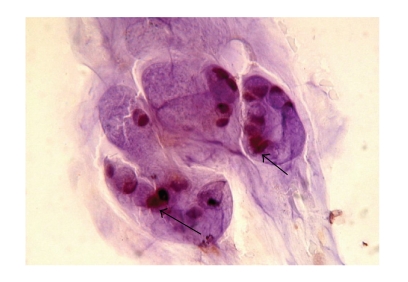
Immunohistochemistry using uPA antibody. Cytologically malignant cells stained positive for uPA cells from cytologic brushings obtained from a pancreatic adenocarcinoma patient during ERCP (arrows).

**Figure 2:**
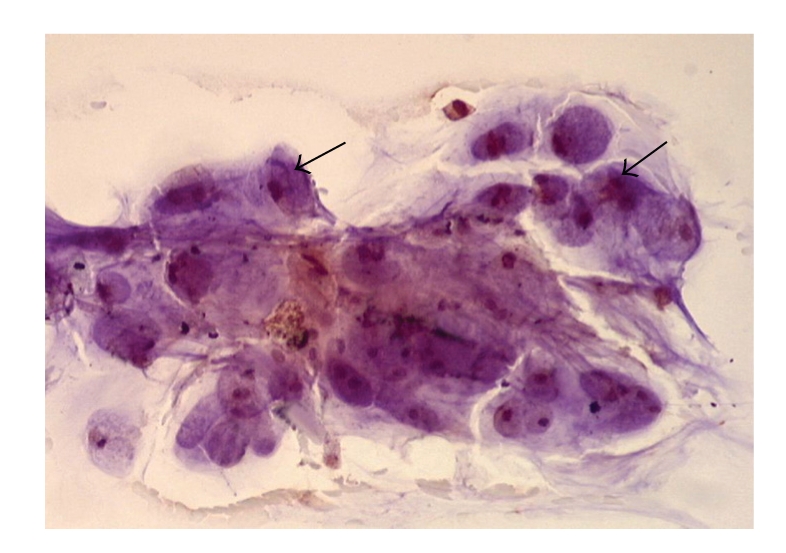
Immunohistochemistry using uPA antibody. Positive staining of cholangiocarcinoma cells from cytologic brushings obtained during ERCP (arrows).

The clinical and pathologic characteristics of patients are summarized in Table 1. There were 8 men and 3 women. The Median age was 61 years (35–82 yrs). Adequate cytologic brushings were obtained in all 11 patients. On final pathology, 8 patients had pancreatic adenocarcinoma and 3 had cholangiocarcinoma. When immunohistochemistry for uPA was performed on the cytology specimens, six of the eight patients (75%) with pancreatic cancer (Figure 1) and two of the 3 patients (67%) with cholangiocarcinoma overexpressed uPA (Figure 2). The two patients with pancreatic cancer who did not overexpress uPA were being treated with chemotherapy and underwent ERCP for biliary stent exchange. The remaining patients were all chemotherapy naïve at the time of biliary decompression.

The mean serum uPA in the 11 patients with cancer was 1.27 ng/mL (SD = 1.54). The mean serum uPA in the 11 normal healthy individuals was 0.56 ng/mL (SD = 0.16). Serum analysis demonstrated a 2-fold higher concentration of uPA in the pancreaticobiliary cancer patients compared to the healthy controls (*P* = .0182). Table 1 compares the level of uPA staining in cytologic specimens to serum levels of uPA for all 11 patients.

The IHC grade for uPA staining correlated with serum levels for uPA (*r* = 0.72; *P* < .0001).

## 4. Discussion

A definitive diagnosis of cancer can be difficult in patients with pancreaticobiliary strictures with no obvious mass on imaging studies. Furthermore patients with an indistinct small pancreatic mass or chronic pancreatitis provide diagnostic dilemmas. Current tumor markers such as CA19-9 and CEA are often not useful. We have previously shown that uPA is an early event in the malignant transformation of pancreatic cancer [15]. Therefore we sought to determine the feasibility of determining uPA expression in cytologic brushings of established malignant strictures to evaluate its potential as an adjunct to ERCP in diagnosis of pancreaticobiliary malignancies. 

In our small sample, we found it is possible to identify overexpression of uPA in cells obtained from brushings during ERCP. uPA overexpression (72.7%) appears to be a marker for pancreatic or biliary cancers. Six out of 8 (75%) pancreatic cancer patients overexpressed uPA. The two patients who did not were being treated with chemotherapy. Although there is no current literature to show that chemotherapy directly inhibits uPA synthesis, we have postulated that this treatment impedes growth, and thus decreases uPA expression. The relationship between uPA overexpression and cholangiocarcinoma has not been studied, but in two of the three (66%) samples, the immunohistochemical analysis of the cytologic brushings showed overexpression. As ERCP is an invasive technique with potential morbidity, we also looked at serum uPA in our patient population, and it was elevated in patients with pancreaticobiliary cancer. The serum levels correlated with the IHC grade of uPA in tissue (*P* < .001). Serum uPA levels have been evaluated in breast cancer by Rha et al., who showed blood uPA levels correlated with those of tissue [22]. Because our study contains only a small number of patients, the value of serum uPA as a tumor marker needs to be further evaluated in large numbers of patients including patients with chronic pancreatitis before it can be compared to the standard tumor markers such as CA19-9.

Currently there are no established screening tests for pancreatic cancer. The United States Preventive Services Task Force (USPSTF) has recommended against routine screening for pancreatic cancer based upon the lack of data demonstrating its clinical value in the general population [23]. Familial pancreatic cancer (FPC) accounts for up to 10% of patients with pancreatic cancer [24]. The precursor lesion to pancreatic cancer is pancreatic intraepithelial neoplasia (PanIN). PanIN is graded from I–III, and patients with PanIN III have been considered for pancreatectomy by some groups [25]. Surveillance by ERCP and endoscopic ultrasound is performed in an attempt to identify patients with FPC who have developed PanIN, but the diagnosis is difficult [26]. We have demonstrated that uPA is overexpressed in 30%–70% of patients with PanIN [15]. Evaluating uPA overexpression from ERCP obtained cytologic brushings may have potential application in screening high-risk patients by identifying either PanIN or invasive pancreatic cancer.

Although ERCP arguably remains the golden standard for pancreaticobiliary evaluation, there is a risk of complications associated with ERCP including pancreatitis (5.4%) [27]. Endoscopic ultrasound (EUS) with EUS-guided FNA has emerged as an extremely sensitive and specific method of diagnosing pancreatic cancer and can detect small lesions more effectively than conventional imaging. For the diagnosis of pancreatic cancer, the sensitivity has been reported to range from 75%–90% and specificity from 82%–100% [28]. As we have shown that uPA staining can be performed on brush cytology, it can be done on FNA specimens as well and may improve diagnostic accuracy.

As targeted therapy has stepped to the forefront of cancer research, molecular markers have moved from their traditional roles of diagnosis and staged to possible aims of treatment. Already uPA has been evaluated as a potential target for treatment to decrease the invasive and metastatic activity of pancreatic tumor cells. Bauer et al. evaluated an anti-uPAR monoclonal antibody in mice and found that it significantly decreased tumor growth, hepatic metastases, and retroperitoneal invasion [29]. Small molecule inhibitors of uPA have been evaluated in a fibrosarcoma model in mice resulting in decreased metastases and prolonged survival [30]. These results suggest that detecting overexpression of uPA in pancreatic cancers may not only be prognostic and diagnostic but may also direct treatment in the future.

A serious limitation of the current study is its small sample size and lack of an adequate control group precluding meaningful analysis. In this study, a control group of healthy subjects for the analysis of uPA in the serum was obtained. There are obvious ethical issues in obtaining brushing from healthy volunteers and it would appear more suitable to utilize patients with benign pancreatic and biliary strictures. We elected to exclude potential control groups such as those presenting with obstructive jaundice from benign pancreaticobiliary strictures. The possibility that some of these patients harbored occult malignancy would be difficult to discern without long term follow up. Even in patients with choledocholithiasis, there may be a potential for the associated inflammatory process to elevate uPA levels. Thus, we performed this feasibility study in patients with known or suspected pancreaticobiliary malignancies by brush cytology to corroborate our prior findings in resected pancreatic cancer specimens.

## 5. Conclusion

We performed a feasibility study to determine whether uPA overexpression could be identified from cytologic brushings of patients undergoing ERCP for confirmed or suspected pancreaticobiliary cancers. Serum levels of uPA were measured in this patient group and correlated with uPA overexpression. Larger studies involving all forms of pancreaticobiliary pathology need to be performed before uPA overexpression can be used either as a serum or a cytological tumor marker, especially those with no identifiable masses. Also comparison with other tumor markers, that is, CA 19-9 and CEA will be required to substantiate the role of uPA as a diagnostic or prognostic tumor marker.

## Figures and Tables

**Table 1:** Clinical and pathologic characteristics of patients with pancreaticobiliary malignancy. IHC staining grade for uPA and serum uPA values for 6 of 8 patients with pancreatic adenocarcinoma and 2 of 3 patients with cholangiocarcinoma overexpressed uPA on cytology obtained from ERCP brushings. Serum values correlated to intensity of staining.

Patient	Sex	Age (yrs)	Cancer Type	Tumor Size	CA 19-9 (U/mL)	IHC Grade	Serum uPA (ng/mL)
1	M	57	Pancreatic	3 cm	27.8	2+	1.18
2	M	35	Pancreatic	4.5 cm	348	3+	0.84
3	F	73	Pancreatic	5.2 cm	45.7	3+	5.86
4	M	71	Pancreatic	3.3 cm	450	2+	0.87
5	M	60	Pancreatic	3 cm	83.9	2+	0.2
6	M	39	Pancreatic	5 cm	126	2+	0.61
7	F	78	Pancreatic	3.0	<1.0	0	0.61
8	F	56	Pancreatic	4 cm	138	0	0.53
9	F	78	Cholangiocarcinoma	1.2	42	3+	0.98
10	M	82	Cholangiocarcinoma	1.0	830.3	2+	0.73
11	M	78	Cholangiocarcinoma	2 cm	52.3	0	0.52
